# Off-Season Diet and Ecology of the Boll Weevil Influence Long-Term Malathion Susceptibility

**DOI:** 10.3390/insects17050484

**Published:** 2026-05-09

**Authors:** Karolayne L. Campos, Guilherme G. Rolim, Raquel S. Silva, Maria R. S. Soares, Cristina S. Bastos, Jorge B. Torres

**Affiliations:** 1 DEPA-Entomologia, Universidade Federal Rural de Pernambuco (UFRPE), Rua Dom Manoel de Medeiros s/n, Dois Irmãos, Recife 52171-900, Pernambuco, Brazil; karolaynelopescampos@gmail.com (K.L.C.); raquels053@gmail.com (M.R.S.S.); 2 Instituto Mato-Grossense do Algodão, Rua Engenheiro Edgard Prado Arze, 1777 Centro Político Administrativo, Cuiabá 78049-015, Mato Grosso, Brazil; guilhermerolim@imamt.org.br (G.G.R.); raquel2sales@gmail.com (R.S.S.); 3 Faculdade de Agronomia e Medicina Veterinária (FAV), Campus Darcy Ribeiro, Universidade de Brasília (UnB), ICC-Sul, Asa Norte, Brasília 70910-900, Distrito Federal, Brazil; cschetino@unb.br

**Keywords:** *Anthonomus grandis grandis*, Cerrado, insecticide resistance, resistance mitigation, cotton fallow

## Abstract

**Background**: The cotton boll weevil, *Anthonomus grandis grandis* Boheman (Coleoptera: Curculionidae), is a major pest of cotton that causes significant yield losses and costly control measures. As a cotton-specialized species, its population dynamics are heavily influenced by environmental conditions and management practices during the off-season. This study examined the effects of off-season food sources on adult survival, reproductive capacity, and susceptibility to malathion in the last generation before the off-season and in the generation entering the next planting season. **Methods**: Females were fed diets representing off-season conditions (cotton terminals and pollen) and the standard diet (flower buds), and then evaluated for survival, copulation, sperm viability, and egg production. Concentration–mortality bioassays determined malathion susceptibility before and after the off-season. **Results**: Female survival was lowest in those fed cotton terminals, while pollen and flower buds supported higher survival rates. The likelihood of copulation and sperm viability remained similar among diets after 60 days, or up to 100 days in surviving females; however, females fed flower buds had more eggs in their oviducts. Females that mated early maintained viable sperm up to the end of the 100-day assessment period. Populations collected after the off-season were more susceptible to malathion than those collected at the end of the growing season. **Conclusions**: Food restrictions and reduced malathion exposure during the off-season may contribute to a reversion toward boll weevils’ susceptibility to malathion. Overall, the findings highlight the importance of the fallow period and proper management of residual off-season populations for effective long-term boll weevil control, confirming malathion’s continued viability as a management tool.

## Simple Summary

The cotton boll weevil (*Anthonomus grandis grandis*) remains one of the most damaging pests of cotton. Its survival between cultivation seasons can influence population growth and insecticide efficacy. In this study, we examined how different off-season food sources affect adult survival and reproduction, and whether boll weevils entering the next planting season are more susceptible to the insecticide malathion after selection pressure is removed. During the off-season, adult females were fed cotton terminals, pollen, or flower buds. Cotton terminals resulted in the lowest survival rate, whereas pollen and flower buds supported higher survival. Diet did not affect reproductive traits such as copulation or sperm viability; however, females fed flower buds produced more eggs. Boll weevil populations collected after the off-season showed reversion toward susceptibility compared with those collected at the end of the growing season. These findings indicate that lower food availability and reduced insecticide exposure during the off-season may mitigate selection for malathion resistance. Managing residual boll weevil populations during the fallow period is critical for long-term pest control and resistance management in cotton production systems.

## 1. Introduction

The cotton boll weevil, *Anthonomus grandis grandis* Boheman (Coleoptera: Curculionidae), is a pest species specialized in cotton in Brazil. It has a long co-evolutionary history with *Gossypium* species and can persist during the off-season on wild and semi-domesticated cotton species [1], when available [2]. These hosts have limited distribution in Brazil, occurring mainly in restricted areas of the northeastern semi-arid region or in backyard systems, and some are at risk of extinction (e.g., *Gossypium mustelinum*, *Gossypium barbadense*, and *Gossypium hirsutum* var. *marie galante*) [3]. Furthermore, the boll weevil is an invasive species in Brazil, having arrived approximately 40 years ago, with biology and dynamics associated with herbaceous cultivated cotton, *Gossypium hirsutum*, with reproduction taking place on flower buds and bolls. 

In the current Brazilian production systems carried out across the Cerrado areas of Brazil, volunteer cotton plants and regrowth from cultivated fields appear to be the primary sources sustaining reproductive populations between seasons [4], with survival increased by feeding on pollen of Cerrado native plants [5]. As a result, cotton production systems significantly influence population dynamics [3,6,7]. The characteristics of the cotton production system in the Cerrado—such as the abundant food availability, agricultural landscape configuration, and crop seasonality—directly influence the cotton boll weevil’s reproductive success and year-round population maintenance [3,6,7,8,9,10]. Under these food-availability and climatic conditions, the number of cotton boll weevil generations can be estimated from its thermal requirements [11]. It is projected that the boll weevil is able to complete up to 6.6 generations during the cropping season between February and July, averaging 1.09 generations per month. Additionally, the abundant availability of cotton fruiting structures facilitates oviposition and development, promoting generational overlap. Different developmental stages of boll weevils are consistently observed in the field. Moreover, the presence of volunteer cotton plants from August, marking the end of the growing season, through January, marking the beginning of the next planting season, can support up to 7 boll weevil generations based on its thermal requirements. This rate corresponds to an average of 1.19 generations per month, given the warmer months of the year. Shifts in the production system within Mato Grosso’s Cerrado may be responsible for this phenomenon. This area is characterized by a concentration of second-crop planting, specifically in January [12]. The emergence of volunteer cotton plants within the first-cropping season, which begins in September with soybeans and corn, contributes to the persistent presence of the boll weevil in the field [3,6,7,8,9], when a null or minimal population would be expected due to its specialization on cultivated cotton for feeding and reproduction.

In this context, the reproduction of the boll weevil reflects not only the quality and continuity of host availability but also the selective pressures imposed by management practices, such as the repeated use of insecticides in the production system [12,13,14]. The extent of cotton plant availability in post- and pre-cotton crops, which is associated with the presence of the boll weevil during both the growing and off-seasons [3,6], together with environmental influences and management practices, can modulate patterns of dispersion, gene flow, and connectivity between populations across the agricultural landscape, despite the preserved genetic variation observed in cotton boll weevil populations [15,16,17]. The interaction of the environment, reproductive physiology, and lack of insecticide exposure during the off-season can thus be linked to the persistence of the boll weevil’s susceptibility to malathion, particularly in terms of the biological and operational factors involved in resistance mitigation [18]. Understanding these relationships can provide useful information for the long-term management of cotton boll weevils.

Although various management tactics are used, the application of synthetic insecticides remains the most common practice for managing the boll weevil [12,13]. The boll weevil’s biological and behavioral traits make this approach less effective, necessitating multiple applications throughout the cotton-growing season [12,19]. Most producers commonly use broad-spectrum insecticides such as the organophosphate malathion. On average, malathion is applied 7.2 times per growing season [12]. Due to the intensive use of insecticides, there have been documented variations in insecticide effectiveness, including for fipronil [14], and reports of pyrethroid resistance [13]. However, insecticides such as malathion, which have been used for decades, have not shown resistance in cotton boll weevils [13,14,20,21].

As a result, it is critical to understand how off-season environmental variables, particularly the quality of available food, affect boll weevil survival and reproduction, as well as the susceptibility of weevil populations that were exposed to selection pressure during the growing season and those entering the new growing cycle after the off-season. The hypothesis is that while recurrent exposure to malathion during the growing season selects for resistance, biological and ecological forces counteract this selection in the off-season, thereby maintaining population susceptibility. This phenomenon may be driven by reduced reproductive performance and survival resulting from fitness costs associated with resistance [22,23], which may be intensified by poor food quality and exposure to insecticides with different modes of action when populations reproduce on volunteer cotton in soybean and corn fields preceding cotton cultivation. For instance, the Colorado potato beetle individuals exhibit higher overwintering mortality, leading to seasonal declines in resistance frequency [24]. Likewise, field-populations of fruit fly that had evolved insecticide resistance during the growing season had reduced survival and showed patterns of lower resistance in the following season [25]; while reduced plant quality increased fitness costs of resistance and altered survival and development of diamondback moth [26] Accordingly, some of the limitations imposed by the off-season diet on boll weevil reproductive capabilities, survival, and susceptibility to malathion were assessed using individuals from the last generation of the growing season (collected during harvest). Individuals obtained after the off-season were collected from infested fruiting structures of volunteer cotton plants in soybean fields during the maturation period, more than four months after the last cotton harvest and before the subsequent cotton season. Thus, the study evaluated the survival rates of mated females, the viability of sperm from females fed off-season diets (cotton terminals or pollen) compared with those fed flower buds, the presence of eggs at intervals corresponding to the mandatory cotton fallow period, and insecticide susceptibility before and after the off-season. 

## 2. Materials and Methods

### 2.1. Survival and Reproduction Status of Boll Weevils Fed Off-Season Cotton Diets

Cotton bolls exhibiting signs of boll weevil infestation were collected from commercial fields during the harvest period in Campo Verde, Mato Grosso (15°16′37.5″ S and 54°58′31.3″ W). The infested structures were transported to the Entomology Laboratory of Instituto Mato-grossense do Algodão (IMAmt) in Campo Verde, MT, where they were held in rearing cages measuring 60 cm × 60 cm × 90 cm (W × H × L) and covered with polyester mesh. Relative humidity inside the cages was maintained by spraying water regularly on the collected reproductive structures until the insects completed development and adults emerged.

Adults emerging from the cages were separated into males and females by examining the abdomen shortly after emergence. The procedure was performed by gently squeezing the abdomen until the genitalia were exposed, and then the size and curvature of tergum 8 were used to discriminate between males and females [27]. Adults were then placed separately in plastic Petri dishes (90 mm × 5 mm in diam. × H) and fed a diet of pollen, brewer’s yeast, and honey (3:6:8) for four days to achieve sexual maturation. At the end of the sexual maturation period, males and females were paired and held for 48 h to ensure mating, with flower buds offered as food. After the mating period, females were randomly assigned to one of three diets. We considered only females because they are responsible for producing the next generation of weevils infesting cotton fields. Thus, they were placed in 1000 mL plastic pots and fed diets representing off-season food: cotton terminals or pollen, and the standard diet: flower buds. We used honeybee pollen harvested from hives maintained in the region (Casa do Mel, Chapada dos Guimarães, MT) to represent pollen from local wild plants available for weevils during the off-season, according to the broad pollen present in the digestive tracts of weevils collected in the Cerrado [5]. The pollen was offered as granules placed in a small container inside the cage, along with tap water in 2 mL microtubes, sealed with cotton. The flower buds at the developed stage (~8 mm) and cotton plant terminals (similar to cotton regrowth) were collected from plants of the IMA 5801 B2RF cultivar grown in a greenhouse (standard diet). Flower buds and terminals had their petioles fixed to humid flower foam to maintain their turgidity. The insects were reared in the laboratory at 25 ± 2 °C, a 12:12 h (L:D) photoperiod, and an average humidity of 50%.

Due to adults emerging on different days and logistical constraints in sexing and establishing the study, the experiment was conducted in two steps, later treated as experimental groups 1 and 2, with randomization incorporated into the analyses. A total of 340 females were tested in each experimental group. Throughout the study, the diets were replenished at least once a week and whenever food quality or availability was compromised. The mating status of females was checked before assignment to the experiment, and at the 20-day intervals, a sample of females (with a minimum confinement time on the diets of 60 days, corresponding to the fallow period) was examined for reproductive characteristics, and those surviving up to 100 days.

Females were dissected to determine mating status, sperm viability, and the presence of developing eggs in the reproductive system [4]. Briefly, the females were chilled at +5 °C for 10 min and then fixed with an entomological pin number 2 over a layer of solidified paraffin in a Petri dish (9 mm diameter), with 50 µL of saline solution (100 mL of H_2_O, 1 g of NaCl, 0.3 g of CaCl, and 0.1 g of KCl) added [28]. Under a stereomicroscope Model C-leds (Nikon Precision Co., Shanghai, China), with a magnification of up to 3.5 mm × 100 mm, the wings and elytra were removed with tweezers, and the abdomen was gently squeezed to reveal the reproductive system. The organ was then carefully removed from the abdomen using 14 cm straight microsurgical forceps. To check the spermatozoa, the spermatheca was carefully removed. Next, the spermathecae were placed on a microscope slide with a drop of saline solution and gently squeezed with a coverslip to burst and release any sperm. Spermatozoa were identified as viable when they moved and non-viable when they did not, under a 400× optical microscope Model DM 500 (Leica Microsystems, Heerbrugg, Switzerland). When a female is mated and carrying sperm, the spermatheca is yellowish; when empty, it is translucent. To confirm this, we forced the spermatheca apart and looked for sperm [4]. Inspection also looked for developing eggs in the reproductive system.

### 2.2. Female Viable Oviposition Fed Off-Season Diets

Oviposition and egg viability were measured in females at various intervals after they were confined with pollen, flower buds, and cotton plant terminals. In this experiment, 375 adult females were reared with each of the three diets. Thus, after 20, 40, and 60 days on the aforementioned diets, females were individually transferred to 500 mL plastic pots and left for 4 days in contact with at least 5 flower buds (5–8 mm in diameter) collected from pest-free cotton plants cultivated in a greenhouse. The peduncle of each flower bud was inserted into moistened floral foam. Following the oviposition exposure period, the flower buds were retained for another five days and dissected to determine the presence or absence of oviposition, and the presence of newly hatched weevil larvae, which were tallied as viable ovipositions.

Females were dissected after feeding on the three tested diets, and their reproductive state was recorded as a binary response (1 = presence; 0 = absence). Samples were dissected at predetermined intervals to confirm copulation, the presence of viable sperm, and eggs in the female’s oviduct. Likewise, flower buds exposed to females were dissected looking for viable eggs and newly hatched larvae at intervals of 20, 40, and 60 days of confinement on the studied diets, considering the presence of newly hatched larvae as a binary variable, viable (presence of larvae) and unviable ovipositions (absence of larvae).

### 2.3. Susceptibility of Boll Weevils to Malathion

This study evaluated five cotton boll weevil populations. One population served as the susceptibility reference and was reared in the Entomology Laboratory of IMAmt in Campo Verde, MT, and fed an artificial diet [29]. The other populations consisted of adults collected from infested cotton fruiting structures during the harvest period of the growing season, primarily those that emerged from bolls (hereafter named the “before off-season” population). Additionally, adults were collected after the off-season, at the start of the next cotton-growing season. During this period, infested cotton fruiting structures (bolls and flower buds) were collected from volunteer plants found in soybean crops, during the maturation period, prior to the cotton planting date, in the same field locations (hereafter named populations “after off-season” period). These collections were made approximately 140 days after those during the harvest period. The geographical coordinates for the collection sites in Campo Verde, MT, are 15°16′37.5″ S and 54°58′31.3″ W, as well as 15°30′02″ S and 55°02′30″ W. Consequently, these populations can be categorized as the 2022/23 (23 August) and 2023/24 (24 August) harvesting periods, while the population collected after the off-season is referred to as the start of the 2023/24 (24 January) and 2024/25 (25 January) seasons. Field-collected infested reproductive structures were allowed to support adult emergence and were reared until the insecticide assays were completed, as described in the previous experiment.

Adults from all populations were tested separately in concentration-response bioassays using the commercial insecticide malathion (Malathion^®^ EC 1000 g a.i.*L^−1^, FMC Química do Brasil Ltd., Campinas, São Paulo, Brazil), which was diluted in deionized water. The bioassay, designed to determine response curves, was conducted using a completely randomized design, with at least 10 weevils per replicate. For each population, a preliminary test was conducted to establish the concentration threshold for testing, aiming to achieve mortality rates near either zero or 100%. Final tested concentrations were obtained by serial dilution, ranging from 175.5 to 3000 mg a.i.*L^−1^ for populations collected during the harvesting periods and from 50 to 307.5 mg a.i.*L^−1^ for populations collected after the off-season periods, allowing the generation of full concentration-response curves.

Adult weevils 4–7 days old were exposed to the dry residue of malathion obtained on cotton leaf discs approximately 8 cm in diameter, along with flower buds without bracts [13]. These green materials were immersed in the insecticide solution at each concentration. After immersion in either the insecticide solution or water (control), the leaf discs and flower buds were allowed to dry at room temperature. They were then placed in glass Petri dishes measuring 12 cm × 0.9 cm, lined with filter paper. The complete bioassay was conducted three times, with all treatments maintained under laboratory conditions. To assess mortality, tested weevils were placed in glass Petri dishes and positioned on a Hot Plate (Fisatom mod. 752A, Rio de Janeiro, Rio de Janeiro, Brazil) calibrated to 35 °C. This stimulates movement in any boll weevils exhibiting thanatosis. Individuals that remained immobile for one minute under these conditions were considered dead. Conversely, all surviving boll weevils exhibited locomotion after being placed on the warmed plate.

Furthermore, in order to make a historical comparison of boll weevil susceptibility to malathion, data from three previous growing seasons were incorporated (populations before off-season 2019/20, 2021/22, and 2022/23). These data were collected exclusively at the end of the growing seasons using the same collection, rearing, bioassay conduction, and data analysis methodology described in this study. These bioassays were carried out in a continuous, standardized manner using populations from the same region as part of the monitoring program conducted in collaboration with the IMAmt in Campo Verde, MT.

### 2.4. Statistical Analysis

#### 2.4.1. Survival and Reproduction Status of Boll Weevils Fed Off-Season Cotton Diets

The mortality rate of individuals reared and fed cotton terminals, pollen, and flower buds was assessed at 5 time points: 20, 40, 60, 80, and 100 days. Monitoring of 341, 340, and 340 females began at 6 days of age, when they entered the pre-established feeding condition, which included 4 days of sexual development and 2 days of mating. The number of dead (rated as 1) and alive (rated as 0) females recorded at 20, 40, 60, 80, and 100 days of confinement in the diets were used as events of interest in the survival analysis. The survival curves were constructed using the Kaplan–Meier method and compared pairwise with the log-rank test via SAS 9.3 PROC LIFETEST [30].

#### 2.4.2. Female Viable Oviposition Fed Off-Season Diets

The data were analyzed using generalized linear mixed models (GLIMMIX) with a binomial distribution and a logit link function. The model included the predetermined diets (pollen, flower bud, and cotton tips) and assessment times (20, 40, and 60 days) as fixed effects, along with their interactions. The group factor (first and second groups of females), was included as a random effect to account for repeated measures over time and to make inferences about the population. This approach controlled dependence among observations and avoided experimental pseudoreplication. Females in group 1 were tracked for 0 to 100 days, and females in group 2 for 0 to 60 days. However, because the 0–6-day time point is similar across groups and diets, only intervals from 20 to 60 days were examined to improve the reliability of the results. Fixed effects were evaluated using the *F*-test, with degrees of freedom adjusted using the Kenward-Roger approach, which is recommended for mixed models with unbalanced designs (different repetitions across groups and fixed factors). Parameter estimates were obtained using the approximate maximum-likelihood approach (Laplace). The adjusted positive response (1:presence) was computed using LSMEANS and then transformed to the probability scale (using the ilink function), with the standard error and 95% confidence interval reported. Tukey’s test (α = 0.05) was used to compare treatment levels (diets) within and across reproductive data for each diet. All statistical analyses were carried out using SAS software, version 9.3 [30], with the PROC GLIMMIX.

#### 2.4.3. Susceptibility of Boll Weevils to Malathion

Concentration–mortality bioassay data were analyzed using Probit analysis [31] via PROC PROBIT in SAS 9.3 [30] to calculate lethal concentrations. Further, resistance ratios and their 95% confidence limits were determined based on lethal concentrations [32]. Ratios were considered significant if the confidence limits did not include 1.0. In this analysis, the laboratory population served as the reference for susceptibility. Comparisons were made among populations obtained before and after the off-season period and among populations tested before the off-season period, monitored since the 2019/20 growing season.

## 3. Results

### 3.1. Survival and Reproduction Status of Boll Weevils Fed Off-Season Cotton Diets

**Figure 1:**
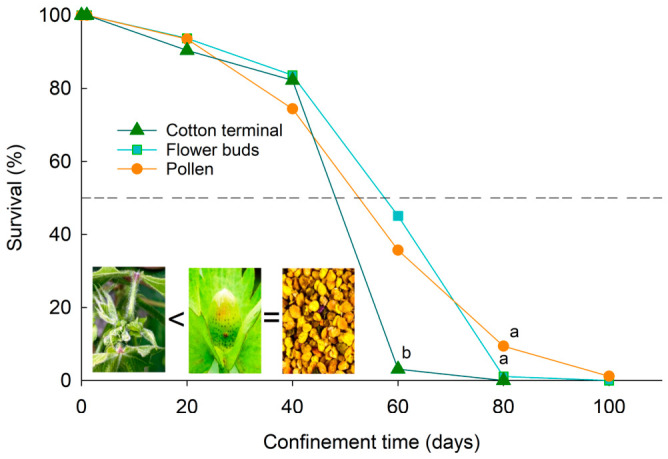
Survival of adult cotton boll weevils, *Anthonomus grandis grandis*, obtained from infested fruiting structures at the harvesting period before off-season and fed cotton terminals, flower buds, or honey bee pollen for up to 100 days in the laboratory. Note: Dashed line indicates the point in time when 50% of the population remained alive; while letters compare curves using the Log-Rank test (α = 0.05).

Adult boll weevil survival was significantly affected by the tested diets (χ^2^ = 86.21; *p* < 0.0001; df = 2; Figure 1). Adults fed pollen or flower buds exhibited similar survival rates (χ^2^ = 1.52; *p* = 0.21; df = 1) and lived longer than those fed cotton terminals (pollen: χ^2^ = 45.85; *p* < 0.0001; flower buds: χ^2^ = 100.33; *p* < 0.0001). However, there was no difference in survival between the diets at 0 to 20 and 40 days across all diets. Adults fed cotton terminals showed a reduced survival rate at 60 days, with no survivors at 80 days (Figure 1), whereas 35.7% and 45.1% remained alive after 60 days when fed pollen or flower buds. The median lethal time (LT50) threshold for females fed flower buds, pollen, or cotton terminals was 56.5, 62.6, and 54.3 days, respectively.

At six days of age, during the sex maturation and pairing period prior to experimentation, 63 females were dissected. These females were fed flower buds, the standard diet, before being confined to the three tested diets, resulting in 59 females that copulated (93.6%). All of these copulated females contained viable sperm in their spermathecae (100%), and eggs were found in the oviducts of 11 females, representing 17.1%.

A total of 245 females from group one (tracked for 0–100 days) and 155 from group two (tracked for 0–60 days) were dissected, yielding 317 individuals for comparison between the groups due to mortality observed over longer time intervals. Given the sample sizes in the two experimental groups, and the different diets, comparisons focused on time intervals of 20, 40, and 60 days, corresponding to the mandatory fallow cotton period. The two and three females that remained alive at the 80- and 100-day intervals when fed pollen, respectively (Figure 1) were fully mated and had viable sperm in their spermathecae; but only 66.7% and 0% had eggs present in their oviducts, respectively. In contrast, eight females fed flower buds survived at 80 days, and four females survived at 100 days; all were 100% mated with viable sperm, and 87.5% of them had eggs present in their oviducts at 80 days, while 50% females had the presence of eggs in their oviducts at 100 days.

In the generalized linear mixed model with a binomial distribution and logistic link, the probability of copulation among females did not show a group effect as a random component (σ^2^ = 0.38 and SE = 0.21; *p* > 0.05), with intercepts (BLUPS) of +0.18 and −0.18 for the first and second groups, respectively. Thus, treatments and periods were treated as fixed factors, with degrees of freedom adjusted using the Kenward-Roger approach. Copulation probability was high (0.85 to 0.99) across all analyzed factors (Table 1), with no significant effect (*p* > 0.05) and only a slight decrease at 60 days. Likewise, females with viable sperm in the spermatheca did not differ by the random group effect (σ^2^ = 0.21, SE = 0.34; *p* > 0.05), with intercepts (BLUPs) of +0.12 and −0.12 (SE = 0.18) for the first and second groups, respectively. Thus, the presence of sperm in the spermathecae followed the same statistical model as the probability of female copulation, with feeding and time as fixed variables in PROC GLIMMIX. The likelihood of females having viable sperm was high (0.89 to 0.96) across all examined parameters (Table 1), with no significant effects of factors or interactions (*p* > 0.05).

Regarding the variable presence of eggs in the female reproductive system, the mixed model indicates a random group effect (σ^2^ = 0.62 ± 0.21; *z* = 2.95; *p* = 0.0032), with BLUP intercepts of −0.41 and +0.41 and SE = 0.18 for the first and second groups, respectively, indicating heterogeneity. Therefore, the analysis retained group as a random factor. Including the random effect improved the fit of the factorial model for assessing intervals and diet (χ^2^ = 7.84; *p* = 0.0051), justifying the use of the generalized linear mixed model with an intraclass correlation coefficient (ICC = 10.8%), which was considered a small effect (ICC within 0.05–0.15) [33]. Thus, interpretation continued with mixed models, focusing on the main factors of interest evaluation intervals and diets. Females with eggs in the reproductive system were significantly influenced by evaluation intervals (Wald χ^2^ = 29.84; df = 2; *p* < 0.0001), offered diet (χ^2^ = 41.12; df = 2; *p* < 0.0001), and the interval and diet interaction (χ^2^ = 9.67, df = 4, *p* = 0.0462). Diet affected initial egg development, as evidenced by differences at 20 days after confinement between females fed pollen and cotton terminals and those fed flower buds (Table 1). There is a delay in egg development, with fewer eggs at 20 days for females fed pollen or cotton terminals, and fewer eggs are maintained in females fed terminals compared to those fed flower buds.

### 3.2. Female Viable Oviposition Fed Off-Season Diets

**Figure 2:**
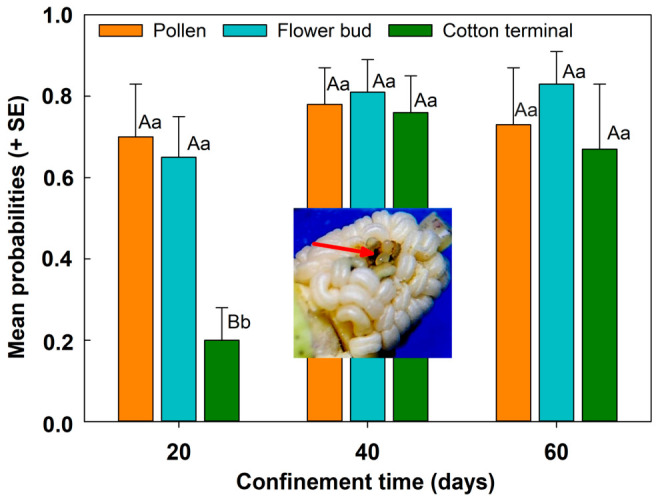
Estimated probabilities (LSMEANS + EP) of viable oviposition (arrow indicates viable egg and larva) found on flower buds exposed during four days to females fed different diets at three intervals of confinement post pairing. Note: Comparisons among treatments within each time interval (capital letters) and within each treatment across time of confinement (small letters), when significant, were performed using Tukey’s honestly significant difference test (α = 0.05).

The number of viable eggs deposited by females varied by diet (F_2,135_ = 4.89; *p* = 0.009) and assessment intervals (F_2,135_ = 6.31; *p* = 0.002), but the interaction between these factors was not significant (F_4,135_ = 1.72; *p* = 0.147). At 20-day intervals of confinement, females fed pollen and flower buds had a significantly higher probability of laying viable eggs than those fed cotton terminals (Figure 2). At 40- or 60-day intervals, however, no significant differences were detected across treatments, indicating that oviposition probability had converged over time. Overall, the production of females laying viable eggs increased over time up to the 60-day evaluation interval, with 67% to 73% of females laying viable eggs, regardless of diet, indicating that diet and time did not interact significantly.

### 3.3. Susceptibility of Boll Weevils to Malathion

**Figure 3:**
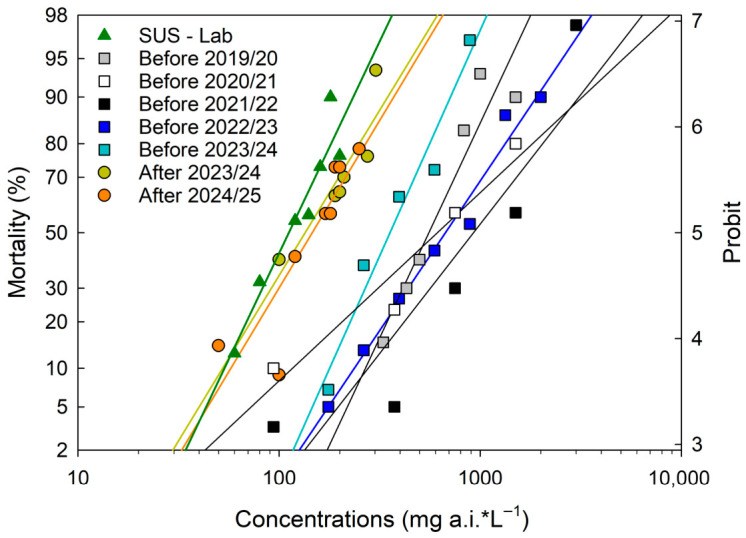
Mortality–concentration curves for populations of the cotton boll weevil (*Anthonomus grandis grandis*) obtained from infested structures collected at the end of the growing season (BEFORE the off-season) of the 2022/23 and 2023/24 seasons and at the beginning of the next (AFTER the off-season) 2023/24 and 2024/25 seasons, with 48 h of exposure to dry malathion residues.

The concentration–mortality responses of all tested populations adequately fit the Probit model (*p* > 0.05), allowing reliable estimation of LC_50_ values (Table 2). Malathion LC_50_ values ranged from 94.22 to 1176.00 mg a.i.*L^−1^. The laboratory population showed the highest susceptibility, with the lowest LC_50_ serving as the reference population for susceptibility (Figure 3—far left). Populations collected after the off-season showed similar levels of susceptibility across the two evaluated seasons (2023/24 and 2024/25), with LC_50_ Values of 139.00 and 136.95 mg a.i. L^−1^, respectively, pooled. In contrast, corresponding populations collected at the end of the growing season (before the off-season) exhibited substantially higher LC_50_ values, reaching 667.74 mg a.i.*L^−1^ in the 2022/23 season and 341.97 mg a.i.*L^−1^ in the 2023/24 season (Table 2—far right). Based on these results, populations obtained before off-seasons 2022/23 and 2023/24 showed resistance ratios (RR_50_) of 7.09 and 3.63 relative to the laboratory reference population (Lab-SUS RR_50_F). Compared with their corresponding after off-season populations entering the subsequent growing season, resistance ratios (RR_50_P) were 4.85 and 5.09, respectively (Table 2). 

Monitoring malathion susceptibility in boll weevil populations at the end of the growing season (before the off-season) from each cropping season between 2019/20 and 2024/25 showed that LC_50_ values consistently exceeded those of the susceptible laboratory reference population (SUS-Lab) and of populations evaluated after the off-season (2023/24 and 2024/25) (Table 2). The population obtained before the off-season 2021/22 exhibited the highest RR_50_ (ca. 12.48-fold), indicating a substantially reduced susceptibility relative to both SUS-Lab and the after-off-season populations. In contrast, populations evaluated after the 2023/24 and 2024/25 off-seasons showed a marked decline in RR_50_ values (Table 2), reflecting variability of selection pressure during the off-season.

## 4. Discussion

The findings show that the off-season diet plays a measurable role in both the survival and reproductive performance of the boll weevil, with downstream consequences for the population’s susceptibility to malathion. By integrating a laboratory diet study with a malathion susceptibility bioassay using populations collected before and after the off-season, our results provide mechanistic support for the hypothesis that boll weevil biology and the relaxation of malathion selection pressure during the off-season contributed to the seasonal modulation of malathion susceptibility.

Adult survival differed markedly among diets, indicating that off-season food quality is a key determinant of population persistence. The higher longevity observed in females fed pollen or flower bud suggests that these food sources can sustain adults throughout much of the legally mandatory fallow period. This aligns with findings from different regions regarding pollen use by boll weevils [1,2,5], including the Cerrado flora, local to this study, from which have been found weevils feeding on pollen from a variety of botanical species [5]. The rapid mortality observed on cotton terminals indicates that regrowth tissues alone are unlikely to maintain populations for extended periods. Importantly, LT_50_ estimates (≈54–63 days) closely overlapped with the mandatory host-free period of 60 days and no individuals surviving beyond 80 days. These findings strongly support the effectiveness of the host-free period to manage boll weevil populations when combining destruction of crop residues and elimination of volunteer plants. Therefore, strict adherence to residue management and host-free regulations is essential to prevent population carryover and reduce early-season infestations.

Despite clear dietary effects on survival, copulation frequency and sperm viability remained consistently high across treatments. The data imply that copulation in the early adult stage of the cotton boll weevil preserves viable sperm, thereby enabling the deposition of viable eggs. In contrast, dietary constraint has a greater impact on egg production. This finding indicates that early-adult mating and sperm storage in the spermatheca are relatively robust to nutritional stress. From an ecological standpoint, this resilience may allow females that survive the off-season on suboptimal foods to rapidly resume reproduction once high-quality cotton structures become available [34]. Nevertheless, the reduced egg production in females fed pollen or cotton terminals indicates a nutritional bottleneck at the level of vitellogenesis rather than mating success [34]. Thus, diet quality appears to regulate reproductive output primarily through egg maturation rather than through mating limitation. Despite the boll weevil specialization on cotton and the expected population decline during the off-season due to the adoption of the fallow period [35,36], the availability of alternative foods and cotton volunteer plants has allowed adults from the previous season to survive. Consumption of pollen from various sources, as well as other cotton plant structures, is thought to increase individual survival during the off-season. Furthermore, adults emerging from the bolls of the last generation have larger body sizes and greater lipid reserves than those emerging from flower buds, enabling them to survive even under poor conditions [35,37,38].

The use of cotton regrowth shoots and volunteer cottons as food during the off-season underscores the importance of complete crop residue destruction [8,12,37,39], a challenge producers list as one of the most difficult for boll weevil management [8]. Furthermore, the presence of flower buds promotes the survival and reproduction of the cotton boll weevil population throughout the production system [4,8], resulting in individuals with high reproductive potential. Maintaining remnant populations from the previous harvest during the off-season can exacerbate the persistence of resistance-selected individuals in future generations. On the other hand, the adverse conditions imposed by the fallow period, together with the potential adaptive costs of resistance, might reduce this selection process and should be seriously considered in management decisions such as crop rotation, volunteer plant removal, and regrowth elimination.

The laboratory population had the lowest LC_50_, indicating no selection pressure from insecticides and making it a sensitive-reference population [40,41]. The susceptibility bioassays revealed a consistent pattern of higher LC_50_-values in populations collected at the end of the growing season (after selection pressure) than in those collected after the off-season. While this pattern is consistent with seasonal relaxation of selection pressure, the results should be interpreted as evidence of phenotypic reversion toward susceptibility, rather than definitive proof of resistance instability. Nevertheless, the magnitude and repeatability of the seasonal shift across years strongly suggest that ecological conditions during the off-season reduce the frequency of less-susceptible individuals in field populations. This pattern, following the off-season, when there is no selection pressure, can be explained by a decrease in the frequency of resistant individuals, which could be linked to reduced exposure to the same insecticide mode of action [18,40].

One plausible explanation for the observed seasonal pattern is likely the presence of fitness costs associated with reduced malathion susceptibility [40,41,42,43]. Under conditions of limited or poor-quality food during the off-season, individuals carrying resistance-associated traits may experience disproportionately lower survival or reproductive success [43], which needs further investigation. Although fitness costs were not directly measured in the present study, this hypothesis is consistent with theoretical expectations and with reports in other insect–insecticide systems [43,44,45]. Studies quantifying life-history trade-offs in late-season emerging weevils would be particularly valuable for explicitly testing this mechanism.

Malathion was first used in 1956 to control a variety of urban and agricultural pests [46]. In agriculture, it is the primary insecticide for managing the cotton boll weevil [12,47], having played a critical role in its eradication in the United States and in its management elsewhere. Despite widespread use in North American eradication programs, there have been no reports of malathion resistance; only variations in susceptibility have been observed [48], a pattern similar to that observed in Brazil [13,14,49]. In Brazil, malathion is registered for use against various crop pests, including some pest species of soybean and corn crops [50]. However, its primary application is in cotton, particularly for controlling the cotton boll weevil. This approach limits the pest’s exposure to the insecticide outside the growing season, reducing selection pressure and contributing to control sustainability. A similar method was successfully used in Australia to mitigate bollworm resistance to pyrethroids by limiting pyrethroid applications to a single crop per year [51].

From a management perspective, the results reinforce the strategic importance of maintaining a strict and effectively enforced host-free period. Even partial survival of nutritionally stressed females could allow a rapid population rebound once cotton becomes available. Therefore, rigorous destruction of volunteer plants, suppression of regrowth, and coordinated regional monitoring remain essential components of resistance management programs. The continued absence of documented field resistance to malathion in Brazilian populations may, in part, reflect the combined effects of ecological bottlenecks during the off-season and the use of malathion primarily on the cotton crop. In addition, the recommended field rates of malathion for spraying cotton fields against boll weevils range from 10,000 to 20,000 mg a.i.*ha^−1^, which is largely higher than any LC_90_ observed across all populations in this study when diluted into 150 L of spray volume. This indicates that weevils have been repeatedly exposed to high doses of malathion, which likely delayed the selection of resistance by eliminating heterozygous individuals [18,52].

## 5. Conclusions

Off-season tested diets influence cotton boll weevil survival and reproductive output. Female survival varied by diet, with those fed pollen and flower buds living longer than those fed cotton terminals. Despite a decline in overall survival over time, the median lethal time for females across all three diets overlaps with the mandatory 60-day host-free interval, while no survivors were found after 80 days for weevils limited to cotton terminals. These findings demonstrate that rigorous control of cotton regrowth and volunteer cotton plants can be effective against boll weevil persistence. These results also show that strict compliance with host-free period rules, extending to their maximum possible duration, becomes effective for managing boll weevils’ residual populations.

Mating success and sperm viability remained high across all diets, suggesting that females that mated early may retain viable sperm for extended periods. Diet quality affected egg production, with females fed flower buds producing more eggs than those fed pollen or cotton terminals. Viable oviposition was observed after confinement periods during the necessary cotton fallow period, implying that females surviving the off-season may still reproduce when suitable host structures become available.

Malathion tolerance was consistently higher in populations collected at the end of the growing season than those collected after the off-season. Lower LC_50_ values post-off-season indicate a seasonal increase in susceptibility, potentially due to reduced selection pressure during the off-season, which imposes ecological costs on less resistant individuals under unfavorable nutritional, host, and climatic conditions.

Possible constraints to broader application of these results include variability in pollen composition used as a food source depending on the region and behavioral changes when comparing field and laboratory trial results, especially concerning physical conditions and mixed diet available in the field. Nonetheless, these questions open an avenue for future research.

## Figures and Tables

**Table 1:** Adjusted probability (LSMEANS ± SE and 95% confidence interval) of the assessed characteristics for cotton boll weevil females reared on three different diets and confined for 20, 40, or 60 days on the specified diet, following emergence from infested structures collected at end of the growing season (before off-season) pooled group 1 and 2 regarding 2022/23 and 2023/24 seasons, in Campo Verde, MT, and maintained at 25 °C with a photoperiod of 12:12 h (L:D).

Characteristics/ Diets ^1^	Days Confined on Specified Diet (Number of Females Dissected)		
	20 (127)	40 (104)	60 (86)
Copulated females			
Flower buds	0.96 ± 0.02 a A (0.92–0.98)	0.95 ± 0.03 a A (0.86–0.99)	0.94 ± 0.04 a A (0.84–0.98)
Pollen	0.95 ± 0.03 a A (0.87–0.99)	0.92 ± 0.04 a A (0.82–0.98)	0.85 ± 0.06 a A (0.71–0.94)
Cotton terminals	0.99 ± 0.01 a A (0.95–1.00)	0.88 ± 0.05 a A (0.76–0.96)	0.97 ± 0.03 a A (0.88–0.99)
Females with viable spermatozoa			
Flower buds	0.95 ± 0.04 a A (0.88–0.98)	0.95 ± 0.073 a A (0.88–0.98)	0.98 ± 0.01 a A (0.94–0.99)
Pollen	0.91 ± 0.04 a A (0.82–0.96)	0.97 ± 0.03 a A (0.92–0.99)	0.92 ± 0.04 a A (0.83–0.97)
Cotton terminals	0.96 ± 0.02 a A (0.91–0.99)	0.89 ± 0.05 a B (0.77–0.95)	0.90 ± 0.05 a A (0.79–0.96)
Females with eggs in the reproductive system			
Flower buds	0.81 ± 0.06 a A (0.61–0.84)	0.87 ± 0.05 a A (0.67–0.88)	0.90 ± 0.06 a A (0.57–0.98)
Pollen	0.27 ± 0.05 b C (0.14–0.39)	0.39 ± 0.07 ab C (0.30–0.58)	0.58 ± 0.08 a B (0.35–0.70)
Cotton terminals	0.36 ± 0.06 b B (0.27–0.55)	0.63 ± 0.06 a B (0.59–0.83)	0.71 ± 0.07 a AB (0.57–0.78)

^1^ Lowercase letters (horizontal rows) compare diets across time intervals, whereas uppercase letters (vertical columns) compare each reproductive characteristic among diets by Tukey’s test (α = 0.05).

**Table 2:** The relative susceptibility of boll weevils from different origins, depending on the phenology of the cotton ecosystem before and after the off-season, when exposed to dry malathion residues. The chi-square test (χ^2^) was used to assess goodness-of-fit of the Probit model.

Populations Off-Season	N (df) ^1^	Slope (±SE)	LC_50_ (FL_95%_) ^2^	RR_50_L (IC95%) ^3^	RR_50_P (IC_95%_) ^4^	LC_90_ (FL_95%_) ^3^	χ^2 *p*-Value^
SUS–Lab^(F7)^	182 (5)	3.94 (±0.64)	94.22 (77.95–107.15)	-	-	199.32 (169.99–260.99)	4.80^0.44^
After 2023/24	124 (6)	3.53 (±0.63)	139.00 (111.63–163.74)	1.48 (1.27–1.72) *	-	320.65 (257.78–475.01)	5.77^0.44^
After 2024/25	120 (6)	3.31 (±0.73)	136.95 (111.43–162.54)	1.45 (1.18–1.79) *	-	333.90 (251.45–644.76)	5.93^0.42^
After pooled	244 (11)	3.43 (±0.47)	137.76 (120.07–154.71)	1.46 (1.28–1.67) *	-	325.31 (270.46–437.39)	7.56^0.75^
Before 2019/20	170 (5)	4.96 (±0.71)	560.55 (491.57−632.18)	5.95 (5.33–6.64) *	4.07 (3.57–4.64) *	1015 (865.82–1306)	6.02^0.30^
Before 2020/21	140 (3)	2.11 (±0.32)	586.93 (443.01–774.78)	6.23 (3.93–9.86) *	4.26 (2.68–6.79) *	2372 (1595–4629)	4.89^0.17^
Before 2021/22	230 (4)	3.84 (±0.53)	1176.00 (995.93–1383)	12.48 (10.64 –14.63) *	8.53 (7.16–10.17) *	2533 (2044–3534)	4.08^0.40^
Before 2022/23	230 (6)	2.97 (±0.33)	667.74 (568.45–781.46)	7.09 (5.88–8.54) *	4.85 (3.97–5.93) *	1805 (1446–2475)	3.33^0.76^
Before 2023/24	161 (4)	3.91 (±0.54)	341.97 (290.72–394.36)	3.63 (3.19–4.14) *	5.09 (4.35–5.95) *	726.28 (603–966.05)	3.16^0.53^

^1^ Number of adults used in bioassays (degrees of freedom); ^2^ LC—concentration (mg of a.i.*L^−1^) that produces mortality 50% or 90%; ^3^ RR—resistance ratio based on LC50 estimates, where RR50L is between the laboratory population and populations obtained after and before off-season periods; ^4^ RR50P is between the populations obtained before off-season and pooled LC50 for populations obtained after off-season, calculated using the Robertson & Preisler [32] method with a 95% confidence interval estimates; * Significant RR for malathion, since the confidence interval does not include the value 1.0.

## Data Availability

The data that support the findings of this study are openly available in Mendeley data: https://data.mendeley.com/datasets/664hnvr7xg/1.
